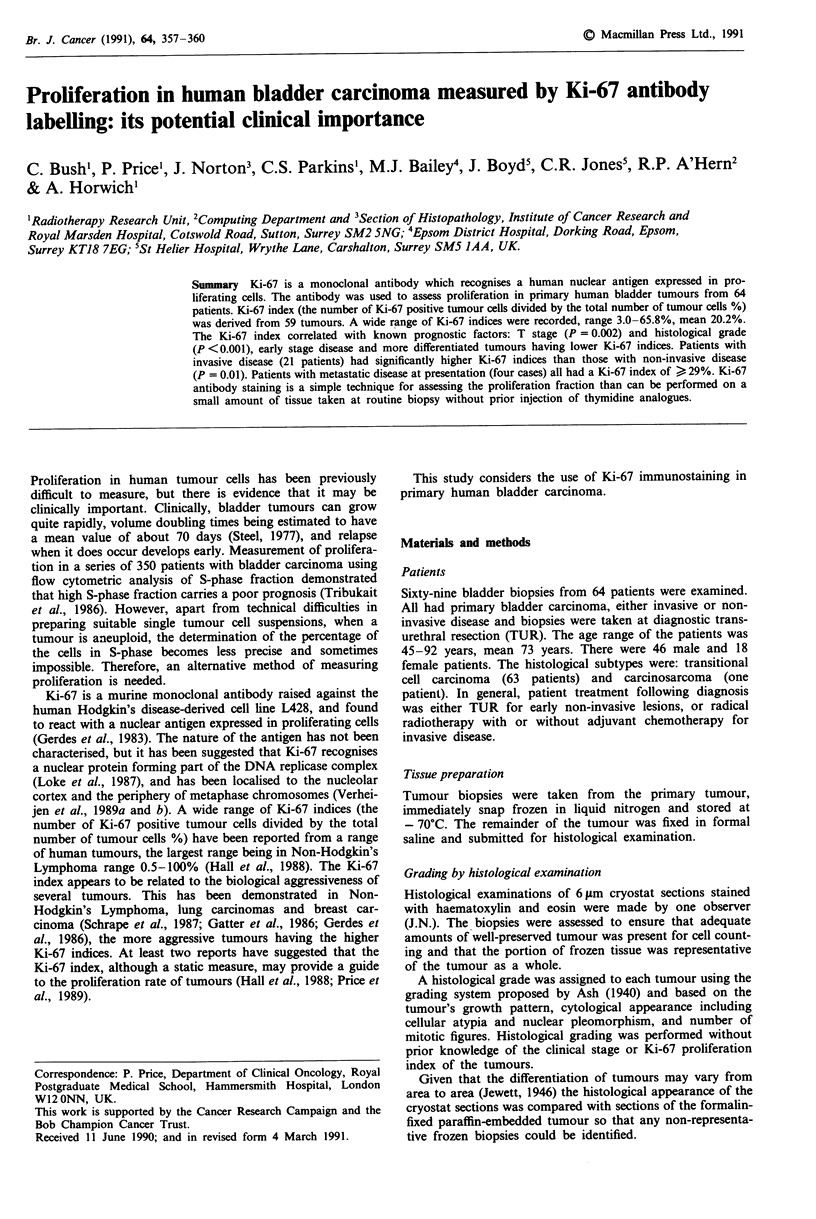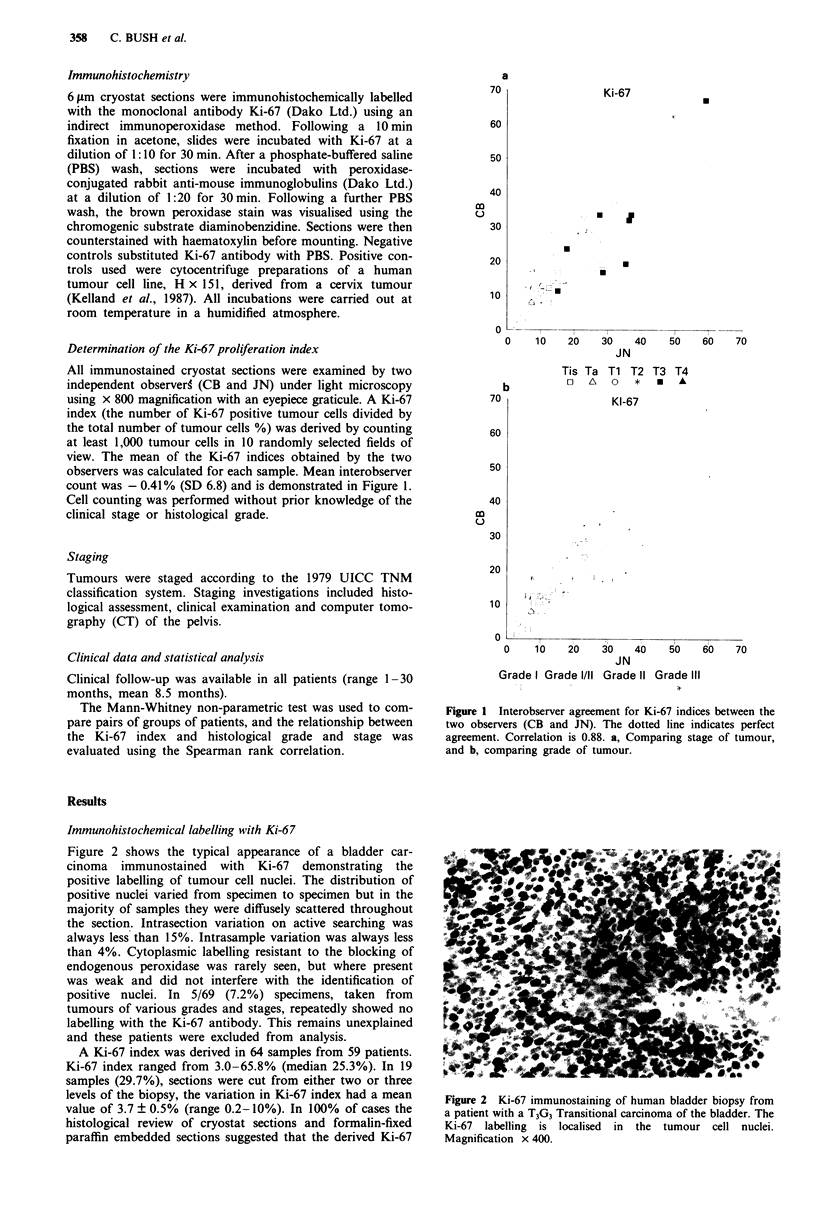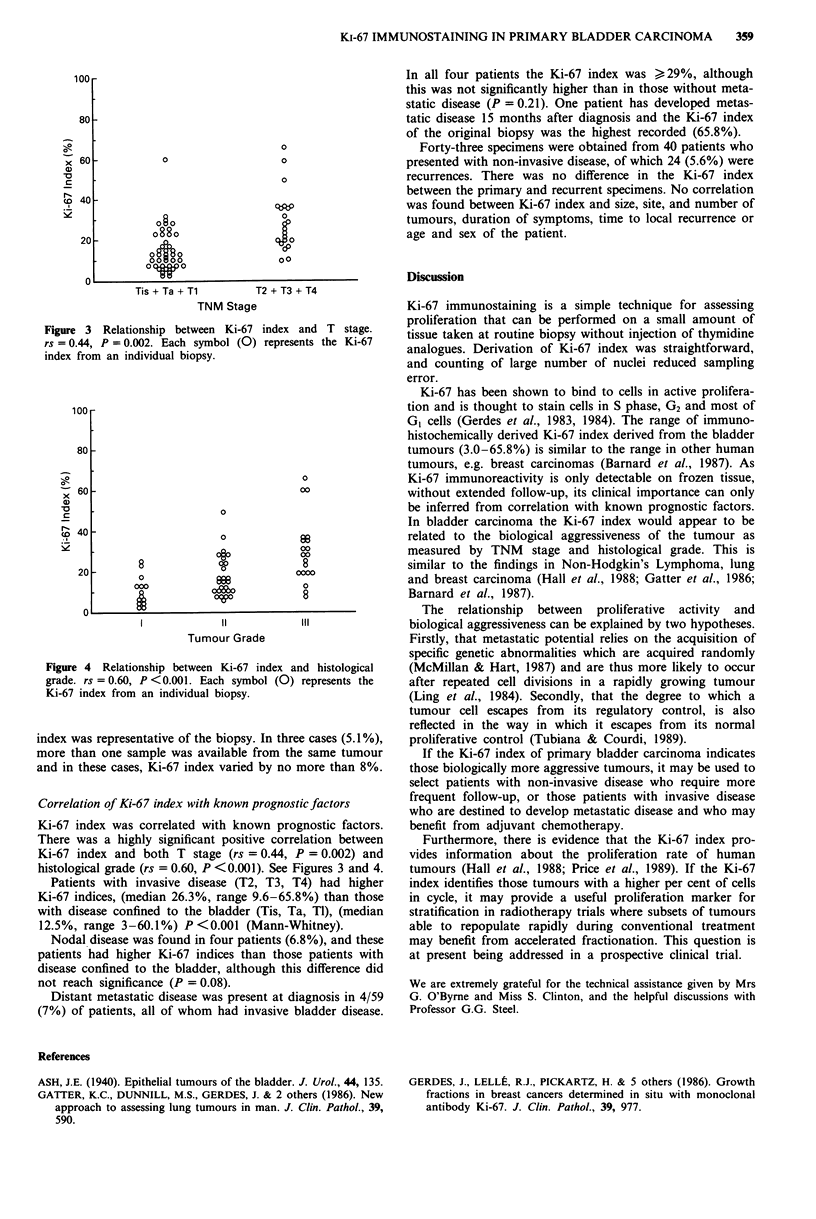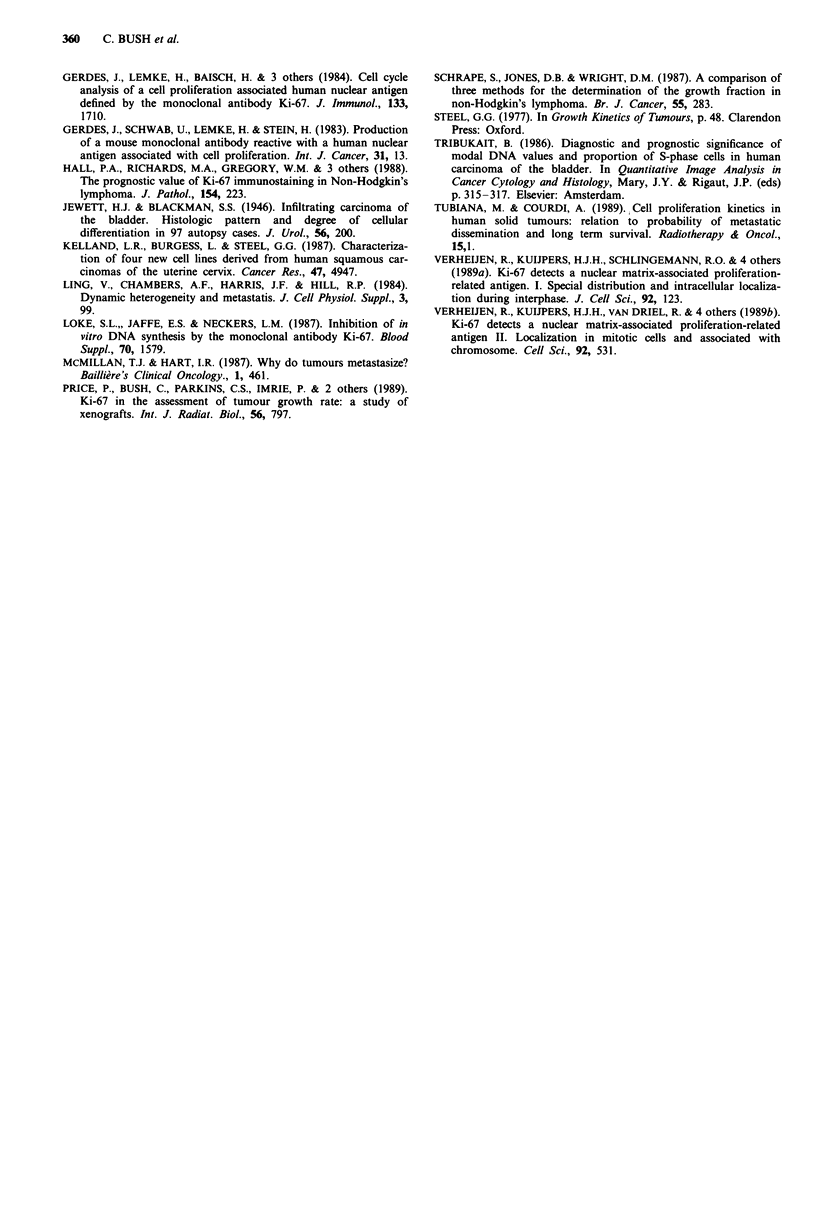# Proliferation in human bladder carcinoma measured by Ki-67 antibody labelling: its potential clinical importance.

**DOI:** 10.1038/bjc.1991.306

**Published:** 1991-08

**Authors:** C. Bush, P. Price, J. Norton, C. S. Parkins, M. J. Bailey, J. Boyd, C. R. Jones, R. P. A'Hern, A. Horwich

**Affiliations:** Radiotherapy Research Unit, Institute of Cancer Research and Royal Marsden Hospital, Sutton, Surrey, UK.

## Abstract

**Images:**


*Br. J. Cancer* (1991), **64**, 357–360 © Macmillan Press Ltd., 1991

### Proliferation in human bladder carcinoma measured by Ki-67 antibody labelling: its potential clinical importance

C. Bush[1], P. Price[1], J. Norton[3], C.S. Parkins[1], M.J. Bailey[4], J. Boyd[5], C.R. Jones[5], R.P. A'Hern[2] & A. Horwich[1]

[1]*Radiotherapy Research Unit,* [2]*Computing Department and* [3]*Section of Histopathology, Institute of Cancer Research and Royal Marsden Hospital, Cotswold Road, Sutton, Surrey SM2 5NG;* [4]*Epsom District Hospital, Dorking Road, Epsom, Surrey KT18 7EG;* [5]*St Helier Hospital, Wrythe Lane, Carshalton, Surrey SM5 1AA, UK.*

**Summary** Ki-67 is a monoclonal antibody which recognises a human nuclear antigen expressed in proliferating cells. The antibody was used to assess proliferation in primary human bladder tumours from 64 patients. Ki-67 index (the number of Ki-67 positive tumour cells divided by the total number of tumour cells %) was derived from 59 tumours. A wide range of Ki-67 indices were recorded, range 3.0–65.8%, mean 20.2%. The Ki-67 index correlated with known prognostic factors: T stage ($P = 0.002$) and histological grade ($P < 0.001$), early stage disease and more differentiated tumours having lower Ki-67 indices. Patients with invasive disease (21 patients) had significantly higher Ki-67 indices than those with non-invasive disease ($P = 0.01$). Patients with metastatic disease at presentation (four cases) all had a Ki-67 index of $\geqslant 29\%$. Ki-67 antibody staining is a simple technique for assessing the proliferation fraction than can be performed on a small amount of tissue taken at routine biopsy without prior injection of thymidine analogues.

Proliferation in human tumour cells has been previously difficult to measure, but there is evidence that it may be clinically important. Clinically, bladder tumours can grow quite rapidly, volume doubling times being estimated to have a mean value of about 70 days (Steel, 1977), and relapse when it does occur develops early. Measurement of proliferation in a series of 350 patients with bladder carcinoma using flow cytometric analysis of S-phase fraction demonstrated that high S-phase fraction carries a poor prognosis (Tribukait *et al.*, 1986). However, apart from technical difficulties in preparing suitable single tumour cell suspensions, when a tumour is aneuploid, the determination of the percentage of the cells in S-phase becomes less precise and sometimes impossible. Therefore, an alternative method of measuring proliferation is needed.

Ki-67 is a murine monoclonal antibody raised against the human Hodgkin's disease-derived cell line L428, and found to react with a nuclear antigen expressed in proliferating cells (Gerdes *et al.*, 1983). The nature of the antigen has not been characterised, but it has been suggested that Ki-67 recognises a nuclear protein forming part of the DNA replicase complex (Loke *et al.*, 1987), and has been localised to the nucleolar cortex and the periphery of metaphase chromosomes (Verheijen *et al.*, 1989*a* and *b*). A wide range of Ki-67 indices (the number of Ki-67 positive tumour cells divided by the total number of tumour cells %) have been reported from a range of human tumours, the largest range being in Non-Hodgkin's Lymphoma range 0.5–100% (Hall *et al.*, 1988). The Ki-67 index appears to be related to the biological aggressiveness of several tumours. This has been demonstrated in Non-Hodgkin's Lymphoma, lung carcinomas and breast carcinoma (Schrape *et al.*, 1987; Gatter *et al.*, 1986; Gerdes *et al.*, 1986), the more aggressive tumours having the higher Ki-67 indices. At least two reports have suggested that the Ki-67 index, although a static measure, may provide a guide to the proliferation rate of tumours (Hall *et al.*, 1988; Price *et al.*, 1989).

Correspondence: P. Price, Department of Clinical Oncology, Royal Postgraduate Medical School, Hammersmith Hospital, London W12 0NN, UK.
This work is supported by the Cancer Research Campaign and the Bob Champion Cancer Trust.
Received 11 June 1990; and in revised form 4 March 1991.

This study considers the use of Ki-67 immunostaining in primary human bladder carcinoma.

### Materials and methods

### *Patients*

Sixty-nine bladder biopsies from 64 patients were examined. All had primary bladder carcinoma, either invasive or non-invasive disease and biopsies were taken at diagnostic trans-urethral resection (TUR). The age range of the patients was 45–92 years, mean 73 years. There were 46 male and 18 female patients. The histological subtypes were: transitional cell carcinoma (63 patients) and carcinosarcoma (one patient). In general, patient treatment following diagnosis was either TUR for early non-invasive lesions, or radical radiotherapy with or without adjuvant chemotherapy for invasive disease.

### *Tissue preparation*

Tumour biopsies were taken from the primary tumour, immediately snap frozen in liquid nitrogen and stored at − 70°C. The remainder of the tumour was fixed in formal saline and submitted for histological examination.

### *Grading by histological examination*

Histological examinations of 6 µm cryostat sections stained with haematoxylin and eosin were made by one observer (J.N.). The biopsies were assessed to ensure that adequate amounts of well-preserved tumour was present for cell counting and that the portion of frozen tissue was representative of the tumour as a whole.

A histological grade was assigned to each tumour using the grading system proposed by Ash (1940) and based on the tumour's growth pattern, cytological appearance including cellular atypia and nuclear pleomorphism, and number of mitotic figures. Histological grading was performed without prior knowledge of the clinical stage or Ki-67 proliferation index of the tumours.

Given that the differentiation of tumours may vary from area to area (Jewett, 1946) the histological appearance of the cryostat sections was compared with sections of the formalin-fixed paraffin-embedded tumour so that any non-representative frozen biopsies could be identified.

*Immunohistochemistry*

6 μm cryostat sections were immunohistochemically labelled with the monoclonal antibody Ki-67 (Dako Ltd.) using an indirect immunoperoxidase method. Following a 10 min fixation in acetone, slides were incubated with Ki-67 at a dilution of 1:10 for 30 min. After a phosphate-buffered saline (PBS) wash, sections were incubated with peroxidase-conjugated rabbit anti-mouse immunoglobulins (Dako Ltd.) at a dilution of 1:20 for 30 min. Following a further PBS wash, the brown peroxidase stain was visualised using the chromogenic substrate diaminobenzidine. Sections were then counterstained with haematoxylin before mounting. Negative controls substituted Ki-67 antibody with PBS. Positive controls used were cytocentrifuge preparations of a human tumour cell line, H × 151, derived from a cervix tumour (Kelland *et al.*, 1987). All incubations were carried out at room temperature in a humidified atmosphere.

*Determination of the Ki-67 proliferation index*

All immunostained cryostat sections were examined by two independent observers (CB and JN) under light microscopy using × 800 magnification with an eyepiece graticule. A Ki-67 index (the number of Ki-67 positive tumour cells divided by the total number of tumour cells %) was derived by counting at least 1,000 tumour cells in 10 randomly selected fields of view. The mean of the Ki-67 indices obtained by the two observers was calculated for each sample. Mean interobserver count was – 0.41% (SD 6.8) and is demonstrated in Figure 1. Cell counting was performed without prior knowledge of the clinical stage or histological grade.

*Staging*

Tumours were staged according to the 1979 UICC TNM classification system. Staging investigations included histological assessment, clinical examination and computer tomography (CT) of the pelvis.

*Clinical data and statistical analysis*

Clinical follow-up was available in all patients (range 1–30 months, mean 8.5 months).

The Mann-Whitney non-parametric test was used to compare pairs of groups of patients, and the relationship between the Ki-67 index and histological grade and stage was evaluated using the Spearman rank correlation.

**Figure 1** Interobserver agreement for Ki-67 indices between the two observers (CB and JN). The dotted line indicates perfect agreement. Correlation is 0.88. **a**, Comparing stage of tumour, and **b**, comparing grade of tumour.

### Results

*Immunohistochemical labelling with Ki-67*

Figure 2 shows the typical appearance of a bladder carcinoma immunostained with Ki-67 demonstrating the positive labelling of tumour cell nuclei. The distribution of positive nuclei varied from specimen to specimen but in the majority of samples they were diffusely scattered throughout the section. Intrasection variation on active searching was always less than 15%. Intrasample variation was always less than 4%. Cytoplasmic labelling resistant to the blocking of endogenous peroxidase was rarely seen, but where present was weak and did not interfere with the identification of positive nuclei. In 5/69 (7.2%) specimens, taken from tumours of various grades and stages, repeatedly showed no labelling with the Ki-67 antibody. This remains unexplained and these patients were excluded from analysis.

A Ki-67 index was derived in 64 samples from 59 patients. Ki-67 index ranged from 3.0–65.8% (median 25.3%). In 19 samples (29.7%), sections were cut from either two or three levels of the biopsy, the variation in Ki-67 index had a mean value of 3.7 ± 0.5% (range 0.2–10%). In 100% of cases the histological review of cryostat sections and formalin-fixed paraffin embedded sections suggested that the derived Ki-67

**Figure 2** Ki-67 immunostaining of human bladder biopsy from a patient with a $T_3G_3$ Transitional carcinoma of the bladder. The Ki-67 labelling is localised in the tumour cell nuclei. Magnification × 400.

**Figure 3** Relationship between Ki-67 index and T stage. $rs = 0.44$, $P = 0.002$. Each symbol (O) represents the Ki-67 index from an individual biopsy.

**Figure 4** Relationship between Ki-67 index and histological grade. $rs = 0.60$, $P < 0.001$. Each symbol (O) represents the Ki-67 index from an individual biopsy.

index was representative of the biopsy. In three cases (5.1%), more than one sample was available from the same tumour and in these cases, Ki-67 index varied by no more than 8%.

*Correlation of Ki-67 index with known prognostic factors*

Ki-67 index was correlated with known prognostic factors. There was a highly significant positive correlation between Ki-67 index and both T stage ($rs = 0.44$, $P = 0.002$) and histological grade ($rs = 0.60$, $P < 0.001$). See Figures 3 and 4.

Patients with invasive disease (T2, T3, T4) had higher Ki-67 indices, (median 26.3%, range 9.6–65.8%) than those with disease confined to the bladder (Tis, Ta, Tl), (median 12.5%, range 3–60.1%) $P < 0.001$ (Mann-Whitney).

Nodal disease was found in four patients (6.8%), and these patients had higher Ki-67 indices than those patients with disease confined to the bladder, although this difference did not reach significance ($P = 0.08$).

Distant metastatic disease was present at diagnosis in 4/59 (7%) of patients, all of whom had invasive bladder disease. In all four patients the Ki-67 index was $\geqslant 29\%$, although this was not significantly higher than in those without metastatic disease ($P = 0.21$). One patient has developed metastatic disease 15 months after diagnosis and the Ki-67 index of the original biopsy was the highest recorded (65.8%).

Forty-three specimens were obtained from 40 patients who presented with non-invasive disease, of which 24 (5.6%) were recurrences. There was no difference in the Ki-67 index between the primary and recurrent specimens. No correlation was found between Ki-67 index and size, site, and number of tumours, duration of symptoms, time to local recurrence or age and sex of the patient.

### Discussion

Ki-67 immunostaining is a simple technique for assessing proliferation that can be performed on a small amount of tissue taken at routine biopsy without injection of thymidine analogues. Derivation of Ki-67 index was straightforward, and counting of large number of nuclei reduced sampling error.

Ki-67 has been shown to bind to cells in active proliferation and is thought to stain cells in S phase, $G_2$ and most of $G_1$ cells (Gerdes *et al.*, 1983, 1984). The range of immunohistochemically derived Ki-67 index derived from the bladder tumours (3.0–65.8%) is similar to the range in other human tumours, e.g. breast carcinomas (Barnard *et al.*, 1987). As Ki-67 immunoreactivity is only detectable on frozen tissue, without extended follow-up, its clinical importance can only be inferred from correlation with known prognostic factors. In bladder carcinoma the Ki-67 index would appear to be related to the biological aggressiveness of the tumour as measured by TNM stage and histological grade. This is similar to the findings in Non-Hodgkin's Lymphoma, lung and breast carcinoma (Hall *et al.*, 1988; Gatter *et al.*, 1986; Barnard *et al.*, 1987).

The relationship between proliferative activity and biological aggressiveness can be explained by two hypotheses. Firstly, that metastatic potential relies on the acquisition of specific genetic abnormalities which are acquired randomly (McMillan & Hart, 1987) and are thus more likely to occur after repeated cell divisions in a rapidly growing tumour (Ling *et al.*, 1984). Secondly, that the degree to which a tumour cell escapes from its regulatory control, is also reflected in the way in which it escapes from its normal proliferative control (Tubiana & Courdi, 1989).

If the Ki-67 index of primary bladder carcinoma indicates those biologically more aggressive tumours, it may be used to select patients with non-invasive disease who require more frequent follow-up, or those patients with invasive disease who are destined to develop metastatic disease and who may benefit from adjuvant chemotherapy.

Furthermore, there is evidence that the Ki-67 index provides information about the proliferation rate of human tumours (Hall *et al.*, 1988; Price *et al.*, 1989). If the Ki-67 index identifies those tumours with a higher per cent of cells in cycle, it may provide a useful proliferation marker for stratification in radiotherapy trials where subsets of tumours able to repopulate rapidly during conventional treatment may benefit from accelerated fractionation. This question is at present being addressed in a prospective clinical trial.

We are extremely grateful for the technical assistance given by Mrs G. O'Byrne and Miss S. Clinton, and the helpful discussions with Professor G.G. Steel.

### References

ASH, J.E. (1940). Epithelial tumours of the bladder. *J. Urol.*, **44**, 135.

GATTER, K.C., DUNNILL, M.S., GERDES, J. & 2 others (1986). New approach to assessing lung tumours in man. *J. Clin. Pathol.*, **39**, 590.

GERDES, J., LELLÉ, R.J., PICKARTZ, H. & 5 others (1986). Growth fractions in breast cancers determined in situ with monoclonal antibody Ki-67. *J. Clin. Pathol.*, **39**, 977.

GERDES, J., LEMKE, H., BAISCH, H. & 3 others (1984). Cell cycle analysis of a cell proliferation associated human nuclear antigen defined by the monoclonal antibody Ki-67. *J. Immunol.*, **133**, 1710.

GERDES, J., SCHWAB, U., LEMKE, H. & STEIN, H. (1983). Production of a mouse monoclonal antibody reactive with a human nuclear antigen associated with cell proliferation. *Int. J. Cancer*, **31**, 13.

HALL, P.A., RICHARDS, M.A., GREGORY, W.M. & 3 others (1988). The prognostic value of Ki-67 immunostaining in Non-Hodgkin's lymphoma. *J. Pathol.*, **154**, 223.

JEWETT, H.J. & BLACKMAN, S.S. (1946). Infiltrating carcinoma of the bladder. Histologic pattern and degree of cellular differentiation in 97 autopsy cases. *J. Urol.*, **56**, 200.

KELLAND, L.R., BURGESS, L. & STEEL, G.G. (1987). Characterization of four new cell lines derived from human squamous carcinomas of the uterine cervix. *Cancer Res.*, **47**, 4947.

LING, V., CHAMBERS, A.F., HARRIS, J.F. & HILL, R.P. (1984). Dynamic heterogeneity and metastatis. *J. Cell Physiol. Suppl.*, **3**, 99.

LOKE, S.L.,, JAFFE, E.S. & NECKERS, L.M. (1987). Inhibition of *in vitro* DNA synthesis by the monoclonal antibody Ki-67. *Blood Suppl.*, **70**, 1579.

MCMILLAN, T.J. & HART, I.R. (1987). Why do tumours metastasize? *Baillière's Clinical Oncology.*, **1**, 461.

PRICE, P., BUSH, C., PARKINS, C.S., IMRIE, P. & 2 others (1989). Ki-67 in the assessment of tumour growth rate: a study of xenografts. *Int. J. Radiat. Biol.*, **56**, 797.

SCHRAPE, S., JONES, D.B. & WRIGHT, D.M. (1987). A comparison of three methods for the determination of the growth fraction in non-Hodgkin's lymphoma. *Br. J. Cancer*, **55**, 283.

STEEL, G.G. (1977). In *Growth Kinetics of Tumours*, p. 48. Clarendon Press: Oxford.

TRIBUKAIT, B. (1986). Diagnostic and prognostic significance of modal DNA values and proportion of S-phase cells in human carcinoma of the bladder. In *Quantitative Image Analysis in Cancer Cytology and Histology*, Mary, J.Y. & Rigaut, J.P. (eds) p. 315–317. Elsevier: Amsterdam.

TUBIANA, M. & COURDI, A. (1989). Cell proliferation kinetics in human solid tumours: relation to probability of metastatic dissemination and long term survival. *Radiotherapy & Oncol.*, **15**,1.

VERHEIJEN, R., KUIJPERS, H.J.H., SCHLINGEMANN, R.O. & 4 others (1989*a*). Ki-67 detects a nuclear matrix-associated proliferation-related antigen. I. Special distribution and intracellular localization during interphase. *J. Cell Sci.*, **92**, 123.

VERHEIJEN, R., KUIJPERS, H.J.H., VAN DRIEL, R. & 4 others (1989*b*). Ki-67 detects a nuclear matrix-associated proliferation-related antigen II. Localization in mitotic cells and associated with chromosome. *Cell Sci.*, **92**, 531.